# Association between benign thyroid disease and breast cancer: a single center experience

**DOI:** 10.1186/s12902-019-0426-8

**Published:** 2019-10-17

**Authors:** Chiara Dobrinja, Serena Scomersi, Fabiola Giudici, Giulia Vallon, Alessio Lanzaro, Marina Troian, Deborah Bonazza, Andrea Romano, Fabrizio Zanconati, Nicolò de Manzini, Marina Bortul

**Affiliations:** 1 Division of General Surgery, Department of Medical and Surgical Sciences, Hospital of Cattinara, University of Trieste, Strada di Fiume 447, 34149 Trieste, Italy; 2 Breast Unit Azienda Sanitaria Universitaria Integrata di Trieste-ASUITS, Division of General Surgery, Department of Medical and Surgical Sciences, Hospital of Cattinara, University of Trieste, Trieste, Italy; 3 UCO Anatomia e Istologia Patologica-Azienda Sanitaria Universitaria Integrata di Trieste-ASUITS, Department of Medical and Surgical Sciences, Hospital of Cattinara, University of Trieste, Trieste, Italy

**Keywords:** Breast cancer, Benign thyroid disease, Thyroid disorders, Menopause

## Abstract

**Background:**

The relationship between breast cancer (BC) and thyroid disease (TD) is still controversial. The aim of the study was to investigate the possible coexistence of TD in patients with newly diagnosed BC and its correlation with BC clinical presentation with regard to menopausal status and stage of disease.

**Methods:**

This is a retrospective cohort study of all patients treated for primary BC between 2014 and 2016 at the Breast Unit of Trieste University Hospital. Clinical charts and reports were reviewed for coexisting thyroid disorders (i.e. hyperthyroidism, hypothyroidism, benign TD, thyroid cancer, thyroid autoimmunity) and menopausal status at the time of BC diagnosis. Biomolecular profile, stage, and grading of BC were also evaluated.

**Results:**

A total of 786 women and 7 men were included in the study. Co-presence of TD was found in 161(20.3%) cases: of these, 151(19.4%) patients presented benign TD and 10(1.3%) patients presented thyroid carcinoma. Thyroid autoimmunity was found in 51(32%) patients. Regarding thyroid function, 88(55%) patients had hypothyroidism, 19(12%) hyperthyroidism, and 54(33%) normal thyroid function. No statistically significant correlation was found between age and TD (*p* = 0.16), although TD was more common in women aged ≥60 years. Women with BC diagnosed at pre-menopausal age were more likely to have thyroid autoimmune diseases (45% vs. 29%, *p* = 0.05). No association was detected among BC molecular profiles with either thyroid autoimmunity (*p* = 0.26) or altered thyroid function (*p* = 0.63). High-grade BC was more frequent in women with hyperthyroidism (52.9%, *p* = 0.04), but the grading was independent from the presence of thyroid autoimmune disease (*p* = 0.87). BC stage was related to both thyroid autoimmunity (p = 0.04) and thyroid function (*p* < 0.001), with 55.2% of women affected by benign TD presenting with stage I BC and more aggressive BCs found in hypothyroid patients.

**Conclusions:**

According our study results, patients with primary BC present a greater incidence of autoimmunity disorders, especially when diagnosed in the pre-menopausal setting. However, further prospective studies are required to definitively prove causality.

## Background

Breast cancer (BC) is the most common malignancy in women and the second most common cancer overall. Over 2 million new cases were diagnosed in 2018, accounting for almost 25% of cancer cases among women [1]. Although hereditary and genetic factors account for 5–10% of BCs, nonhereditary factors are more commonly involved in geographical and ethnic differences in incidence [2–6]. In this context, the relationship of BC with thyroid disease (TD) has been widely investigated. However, data are still controversial and, although almost every form of TD, including autoimmunity disorders and thyroid cancer, has been identified in association with BC, no convincing evidence exists of a causal role for TD in BC [2–6].

A recent meta-analysis performed by Hardefeldt et al. [7] found that patients with autoimmune thyroiditis presented an increased risk of BC (OR 2.92) and subgroup analysis identified a significant association with both anti-TPO and anti-TG thyroid antibodies. Although the exact mechanism linking BC and TD has not been found yet, several hypotheses have been postulated. The possible interactions between thyroid gland and breast tissue may be based on the common property of both epithelial tissues to concentrate iodine by means of a sodium/iodine symporter (NIS), as well as on the presence of TSH receptors in fatty tissue, which is abundant in mammary glands [6]. Alternatively, since estrogen receptors have been identified in abnormal thyroid tissue cells, a reversal in the relationship with BC acting as a trigger for thyroid dysfunction cannot be excluded [3–6]. Additionally, some endocrine stimuli may exert a simultaneous action on both breast and thyroid gland, determining the coincidence of mammary and thyroid disorders [2–7].

Aim of this study was to evaluate the prevalence of TD in a consecutive series of patients treated for primary BC in order to assess any possible association in terms of clinical presentation, management, and oncologic outcome.

## Methods

Between January 2014 and December 2016, 823 patients were treated for BC at the Breast Unit of Trieste University Hospital. Clinical data and information recorded from patients charts as well as radiology and pathology reports were retrospectively reviewed to assess for the following inclusion criteria:

- pathological diagnosis of primary BC confirmed by fine needle aspiration (FNA), core biopsy (CB), or vacuum-assisted biopsy (VAB) with Mammotome®.
- presence of TD, including any form of benign thyroid disease, thyroid cancer, thyroid autoimmunity disorders, and/or altered thyroid function (i.e. hypertyroidism, hypothyroidism).

Patients with recurrent BC, patients with metastatic BC, and patients with histopathological diagnosis of lymphoma or sarcoma of the breast were excluded from the analysis.

According to these inclusion criteria, the study population consisted of 793 patients.

### Data analysis – breast Cancer

Variables routinely documented included patient age, gender, menopausal status for the female sex, BC pathological size, stage, grading and biological profile. In agreement with NCCN Guidelines [8], menopausal status was defined according to women age, with 60 years as a cut-off value.

Immunohistochemical profiling of BC samples was routinely performed as part of clinical care. According to the American Joint Committee on Cancer (AJCC) staging system (7th Edition) [9] and the St. Gallen International Expert Consensus [10], BC was classified into 4 subtypes:

- Luminal A: hormone-receptor (HR) positive, HER2 negative, low levels of Ki-67 protein;
- Luminal B: HR positive, with either positive or negative HER2 and high levels of Ki-67 protein;
- HER2 type: HR negative, HER2 positive;
- Triple negative: HR negative, HER2 negative.

According to the above mentioned classification systems and current treatment guidelines, BC patients underwent surgery (either breast conserving surgery or mastectomy) and/or medical treatment (i.e. preoperative/definitive chemotherapy and/or hormonal therapy).

### Data analysis – thyroid disease

For every patient, the following variables were identified: presence of benign TD, presence and histotype of thyroid carcinoma, presence of autoimmunity TD, thyroid functional status.

Thyroid function was checked in every patient by means of venous dosage of thyroid hormones (i.e. TSH, FT3, FT4) and thyroid antibodies (i.e. anti-TPO and anti-TG).

Hyperthyroidism was defined as high synthesis and/or secretion of thyroid hormones, determining suppressed TSH values (< 0.40 μIU/mL). Hypothyroidism was defined as reduced secretion of thyroid hormones, determining increased TSH values (> 4.00 μIU/mL).

Thyroiditis was defined by the presence of thyroid antibodies and/or by means of ultrasound features and/or on histological examination of surgical specimens. Multinodular goiter was defined by the demonstration of multiple follicular nodules on ultrasound examination and/or on histological examination of surgical specimens.

A thyroid ultrasound was performed in every patient presenting with altered thyroid function. Thyroid scintigraphy was indicated in case of hyperfunctioning disease, whereas FNA was carried out in patients with thyroid nodules suspicious for malignancy. Definitive histological examination was obtained in patients undergoing thyroidectomy.

### Statistical analysis

Quantitative data were reported as mean, median, standard deviation and interquartile range. The qualitative variables were expressed as absolute frequencies and percentages. Chi-square test (or F-Fisher Exact test when appropriate) and Proportion Test were used to assess association between categorical variables:

- Correlation between menopausal status and thyroid disease, autoimmune thyroid disease or thyroid function;
- Correlation between breast cancer biological profile and thyroid disease, autoimmune thyroid disease or thyroid function;
- Correlation between breast cancer staging and thyroid disease, autoimmune thyroid disease or thyroid function;
- Correlation between breast cancer grading and thyroid disease, autoimmune thyroid disease or thyroid function.

Statistical analysis was performed using R (the R Foundation for Statistical Computing; Version 3.0.3) software. A *p* value less than 0.05 was considered statistically significant.

## Results

Between January 2014 and December 2016, 823 patients were referred for BC at the Breast Unit of Trieste University Hospital. Of these, 793 patients were eligible for the purposes of this study.

The cohort consisted of 786 (99%) women (mean age 66 ± 14 years), and 7 (1%) men (mean age 71 ± 12 years). Among the female population, 30 patients presented bilateral BC. Overall, 716 (87%) patients underwent breast surgery and 107 (13%) patients were not considered for surgery because of severe comorbidities and thus medically treated with hormonal therapy. BC molecular profile was reported in 754 (95%) patients, while no information were available in 39 (5%) cases because BC diagnosis was made by on FNA cytology.

Information about thyroid function was recorded in 779 (98%) BC patients. Of these, 161 (21%, 160 women and 1 man) presented both BC and TD, 151 (93.8%) of them showed a benign TD and 10 (6.2%) presented malignant TD. Concerning data for thyroid cancer, mean age at the time of diagnosis was 65 ± 13 years and tumor subtypes were classified as follows: 8 papillary thyroid carcinomas, 1 follicular thyroid carcinoma, and 1 medullary thyroid carcinoma.

Among the 161 BC patients with concurrent TD, 88 (55%) patients had hypothyroidism, 19 (12%) patients showed hyperthyroidism, and 54 (33%) patients had normal thyroid function.

Thyroid autoimmunity was identified in 51 (32%) patients out of 161 cases of BC with concurrent TD. Table 1 shows the distribution of different TDs according to thyroid function.

**Table 1:** Distribution of different thyroid disease divided according to thyroid function

Classification	Diagnosis	Number	%
Hypothyroidism (88 CASES)	Autoimmune	43	49%
	Primary	30	34%
	Multinodular goiter	10	11%
	Caused by amiodarone	2	2%
	Adenoma	1	1%
	Papillary thyroid cancer	1	1%
	After menopause	1	1%
Hyperthyroidism (19 CASES)	Basedow disease	9	47%
	Toxic multinodular goiter	8	42%
	Caused by amiodarone	1	5%
	Medullary thyroid cancer	1	5%
	Plummer	0	0%
Euthyroidism (54 CASES)	Multinodular goiter	24	44%
	Thyroid nodules	21	39%
	Papillary thyroid cancer	7	13%
	Follicular thyroid cancer	1	2%
	Endocrinopathologies not better specified	1	2%

### Correlation between menopausal status and TD, autoimmune TD or thyroid function

Analysis was conducted on a cohort of 754 BC patients. Patients with malignant TD (*n* = 10) and men (*n* = 7) were excluded subgroup analysis.

At first, we investigated the correlation between menopausal status and benign TD. Among pre-menopausal women with BC, benign TD was found in 17% of patients. Similar percentage (21%) was found in BC women aged > = 60 years (*p* = 0.16).

The analysis of correlation between menopausal status and autoimmunity showed that autoimmune TD was diagnosed in 45% of pre-menopausal BC patients and in 29% of post-menopausal BC patients, with a borderline significance at statistical analysis (*p* = 0.05). Moreover, pre-menopausal BC women were more frequently affected by autoimmune TD than post-menopausal BC ones, while benign TD was generally much more common in post-menopausal BC women (71% vs. 29%).

Evaluation of correlation between menopausal status and thyroid function, showed no differences at statistical analysis (*p* = 0.49).

### Correlation between BC biological profile and TD, autoimmune TD or thyroid function

**Fig. 1:**
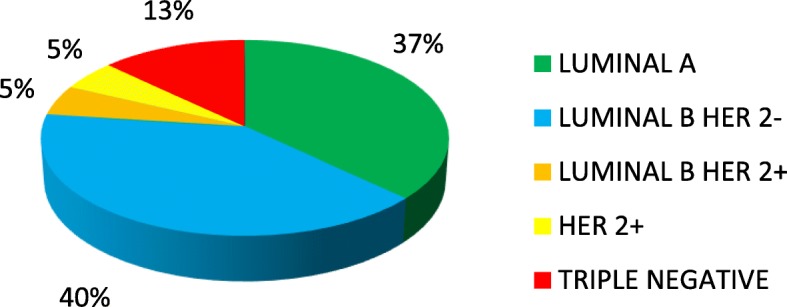
Breast cancer molecular profile

**Fig. 2:**
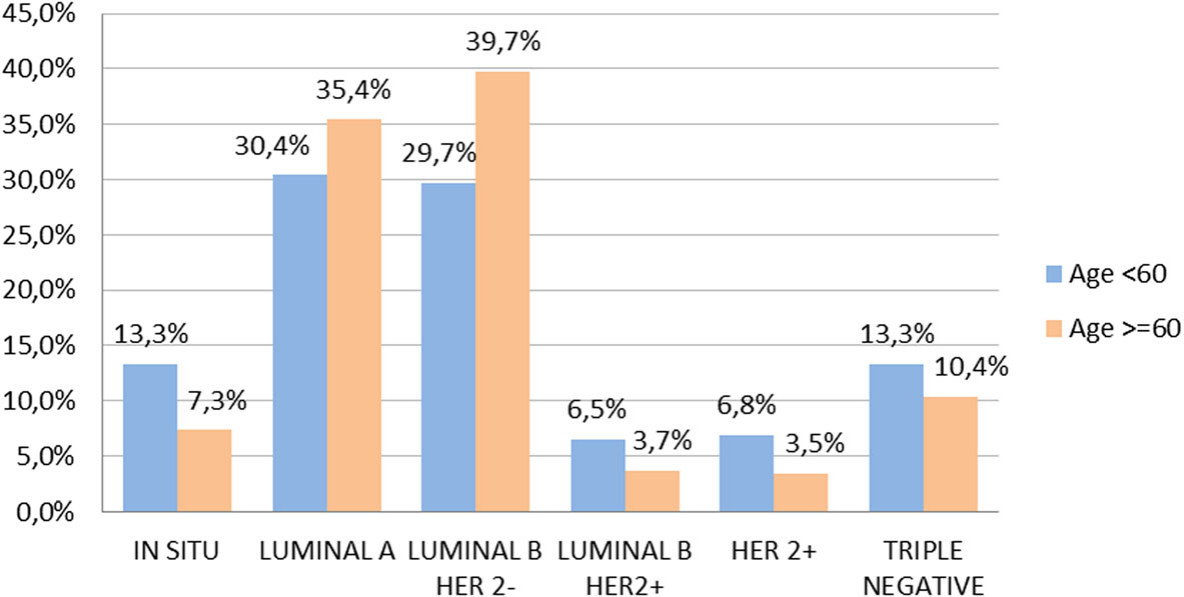
Different breast cancer molecular profiles and their distribution according to menopausal status

Molecular profile of BC was determined on immunostaining for 754 (95%) patients. Different BC molecular profiles and their distribution according to menopausal status are represented in Figs. 1, 2. BC subtypes TN and HER2+ were more frequently observed in pre-menopausal women (*p* < 0.001). BC molecular profile was not associated with benign TD (*p* = 0.85), nor with thyroid autoimmunity (*p* = 0.26) and not even with thyroid function (*p* = 0.63).

### Correlation between BC staging and TD, autoimmune TD or thyroid function

The analysis of a possible correlation between BC staging and benign TD was possible in a subgroup of 136 cases out of 151 (90%), with a median age at the time of diagnosis of 68 years.

BC stage distribution related to benign TD is showed in Table 2.

**Table 2:** Distribution of Breast cancer stage in women with benign thyroid disease

TNM Stage	N°	%
Stage 0	15	11.0%
Stage I	75	55.2%
Stage II	35	25.7%
Stage III	11	8.1%
Total	136	100.0%

Different distribution of BC stage according to thyroid function was recorded (p < 0.001). A major proportion of stage I patients was observed among women with normal thyroid function, whereas patients with higher BC stage were more frequently affected by hypothyroidism (Table 3).

**Table 3:** Breast cancer stage related to thyroid function

Stage	Hypothyroidism	Hyperthyroidism	Euthyroidism
Stage 0	10 (12.2%)	2 (14.3%)	3 (7.5%)
Stage I	42 (51.2%)	6 (42.9%)	27 (67.5%)
Stage II	20 (24.4%)	5 (35.7%)	10 (25.0%)
Stage III	10 (12.2%)	1 (7.1%)	0 (0.0%)
Total	82	14	40

Different distribution of breast cancer stage according to thyroid function (*p* < 0–001, Fisher Test)

Analyzing the presence or absence of autoimmune TD, no difference was found in distribution for stage 0, I and II BC, while stage III BC was much more common among women with autoimmune TD (14.6% vs. 4.5%, *p* = 0,05). Data are reported in Table 4.

**Table 4:** Distribution of breast cancer stage according to the presence or absence of autoimmune thyroid disease

Stage	Absence of autoimmune thyroid disease	Autoimmune thyroid disease
Stage 0	10 (11.4%)	5 (10.4%)
Stage I	50 (56.8%)	25 (52.1%)
Stage II	24 (27.3%)	11 (22.9%)
Stage III	4 (4.5%)	7 (14.6%)
Total	88	48

Breast cancer stage III was more common among women with autoimmune thyroid disease (14.6% vs 4.5%, *p* = 0,05, Proportion Test)

### Correlation between BC grading and TD, autoimmune TD or thyroid function

**Fig. 3:**
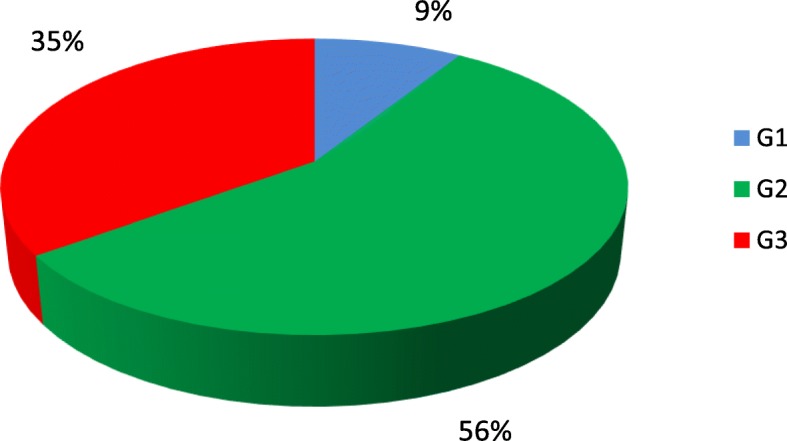
Correlation between breast cancer grading and BTD

Figure 3 shows the correlation between BC grading and benign TD.

Analysis of correlation between BC grading and thyroid function demonstrated that G3 carcinomas were more represented in women with hyperthyroidism (52.9%) than in those with hypothyroidism (38.6%) or normal thyroid function (21.4%) (*p* = 0.04).

BC grading was not related to the presence or absence of autoimmune TD (*p* = 0.87).

## Discussion

BC and TD have a well known epidemiological impact among the world population. Both pathologies have greater incidence in women than in men and an increasing number of cases has been diagnosed in recent years. The possible correlation between BC and TD has been widely discussed in literature, but data remain still controversial [5, 7]. Some studies have shown an increased prevalence of BC in patients with benign TD [4], but a clear relationship between hormonal status and serum thyroid antibodies has not been demonstrated yet [5].

Several hypotheses have been postulated on the mechanism linking TD and BC. Some Authors showed that the absorption and oxidation of iodine may play an important role in the development of BC, which is known having a greater incidence in iodine-deficient geographic areas [11–14]. Another possible interaction may be based on the presence of a sodium/iodine symporter in both the thyroid and breast tissue [15]. In this context, Tazebay et al. [16] demonstrated that there is an increased sodium/iodine symporter expression in thyroid and breast cancer tissues compared to healthy breast tissues. Consequently, this overexpression may play a role in the development of targeted therapies and screening programs for BC. The possible interaction between thyroid and mammary glands can also be explained by the presence of TSH receptors in the adipose tissue of mammary gland. In 2006, Conde et al. [17] showed that pathological samples of non-invasive BCs there were many more thyroid receptors α (TR-α) than in pathological samples of invasive BCs. Furthermore, Jerzak et al. [18] found that BC patients overexpressing this type of receptors showed a better outcome.

The present retrospective analysis aimed to assess the correlation between TD and BC with particular regard to different variables, namely menopausal status, BC molecular profile, and BC staging and grading. According to the results of this study, no differences were observed between pre- and post-menopausal women in terms of benign TD and/or altered thyroid functional status.

Differences was observed regarding autoimmune thyroiditis, with pre-menopausal BC patients being more frequently affected by this pathology than post-menopausal BC patients (45% vs. 29%, *p* = 0.05). Similar results have been recently published by Chiappa et al. [19], who described an association between chronic autoimmune thyroiditis and BC diagnosed in pre-menopausal state. However, these results may not be completely comparable, since for Chiappa and colleagues post-menopause was defined as women aged > = 45 years instead of 60 years. Anyway, several other studies have shown an association between BC and autoimmune TD [20], although the underlying reasons for this correlation are still unclear. In 2001, Gogas et al. [21] demonstrated a higher frequency of autoimmune thyroiditis in subjects with more advanced BC, therefore suggesting a possible correlation between thyroid autoimmunity and a worse BC prognosis. On the other hand, Smith et al. [22] published completely different data, giving evidence of a better prognosis in patients suffering from autoimmune TD. Giustarini et al. [23] evaluated thyroid autoimmunity in patients with malignant and benign breast diseases before surgery and observed a significantly higher presence of thyroid antibodies in BC patients compared to patients suffering from benign breast disease. Recently, Muller et al. [24] found no difference in terms of 10-year survival for BC patients with or without thyroid antibodies at blood tests analysis.

In the present series, the well-known aggressive TN and Her2 positive BC subtypes were more frequently observed in pre-menopausal women (*p* < 0.001), although no correlation was found with thyroid autoimmunity (*p* = 0.26). When considering thyroid functional status, in the current study 55% of patients presented with hypothyroidism, 12% with hyperthyroidism and 33% with normal thyroid function. At statistical analysis, no significant correlation was found with the menopausal status (*p* = 0.49). Regarding the possible relationship between BC staging and the presence of benign TD, this study found that patients with normal thyroid function presented predominantly AJCC stage I disease (67.5%), whereas patients with hypothyroidism presented more frequently AJCC stage II-III disease (12.2%). This could indicate that a reduced thyroid function might predispose to develop a more advanced stage of BC, while a condition of euthyroidism seems to correlate with lower BC stage. In this context, Sogaard et al. [25], in a case-control epidemiological study conducted on a large cohort of Danish women, showed an increased risk of BC in women with hyperthyroidism and a slightly increased risk of BC in women with hypothyroidism. On the other hand, Angelousi et al. [26] reported in their study a lower incidence of lymph node metastasis in hypothyroid patients suffering from BC, thus suggesting that hypothyroidism might represent a protective factor in BC outcome.

As far as the correlation between BC stage and the presence of autoimmune TD is concerned, the results of this study showed that there were no significant differences in the number of patients with autoimmune and non-autoimmune TD in BC stage 0, I and II. Conversely, BC stage III disease was significantly more common among women with autoimmune TD (14.6% vs. 4.5%, *p* = 0,05). This result is surprisingly in contrast with literature data, because several Authors have reported a higher prevalence of thyroid peroxidase antibodies (TPO-Abs) in BC patients, underlining their potentially protective role in terms of oncologic prognosis [27, 28]. Nevertheless, this result may reinforce the hypothesis that a reduced thyroid function, present in most cases of autoimmune thyroiditis, could predispose to develop more advanced stage of BC.

Regarding the concomitant occurrence of BC and thyroid cancer, the present series recorded only 10(1.3%) cases, all women and with a greater predominance of papillary thyroid carcinoma, reflecting the high epidemiological frequency of this type of thyroid cancer.

Last but not least, when assessing for a possible correlation between BC and benign TD, the present analysis showed that the vast majority (56.3%) of women with benign TD had G2 BC while G3 carcinomas were more represented among women with hyperthyroidism (52.9%), suggesting that an increased thyroid function may correlate with a higher grading of BC. Lastly, statistical analysis showed that BC grading is independent from the presence of autoimmune TD.

## Conclusions

The present study has a number of limitation. Being a single-center experience based on retrospective non-randomized analysis, the possibility of generalizing the results is potentially limited. Additionally, the sample size is not very large and the observation period might not be long enough. However, the study managed to confirm that there is a relationship between BC and TD, although further studies with prospective analyses and extended follow-up are required in order to elucidate the nature of this relationship. Future research might investigate the precise pathological mechanisms that correlate the two pathologies also by checking the presence of estrogen and progesterone receptors on histologic specimens of thyroid carcinomas in order to possibly identify a subgroup of patients with higher risk of developing BC.

## Data Availability

Over the period from January 2014 to December 2016, all patients admitted with diagnosis of Breast Cancer were selected and retrospectively analyzed. Data were obtained from electronic database and manual search of studies relatives of Breast cancer and thyroid disease. The datasets used and analyzed during the current study is available from the corresponding author on reasonable request.

## Abbreviations

Ab: Antibodies
BC: Breast cancer
CB: Core biopsy
FNA: Fine needle aspiration
TD): Thyroid disease
TG: Thyroglobulin
TN: Triple negative
TPO: Thyroid peroxidase
TR-α: Thyroid receptors α
VAB: Vacuum assisted biopsy
